# The hidden carbon impact of animal disease

**DOI:** 10.1371/journal.pone.0292659

**Published:** 2023-10-10

**Authors:** Tarek Soliman, Andrew Barnes, Irmelin Slettemoen Helgesen

**Affiliations:** 1 Scotland’s Rural College (SRUC), Edinburgh, United Kingdom; 2 Norwegian University of Science and Technology, Trondheim, Norway; Bangladesh Agricultural University, BANGLADESH

## Abstract

Livestock production is under scrutiny for its impact on greenhouse gas (GHG) emissions. Animal disease outbreaks will have economic effects on producers and the indirect cost of an animal disease outbreak is the result of shifts in consumption across commodities. This shift in demand for meat products will also positively or negatively affect carbon emissions. We explore the indirect costs and subsequent carbon impact of four potential exotic disease outbreaks, namely African swine fever, sheep pox, bluetongue, and foot and mouth disease. The indirect costs are quantified under different severities of outbreak using a vector error correction model and by estimating the changes in revenues of livestock and feed markets. By associating subsequent consumption switches with emission factors, we quantify the hidden carbon impact of these livestock disease outbreaks. The indirect costs vary based on severity and type of disease outbreak. Similarly, the net reduction in supply and subsequent consumption impacts result in averting between 0.005 and 0.67 million tonnes of CO_2_ eq. for these sectors. A foot and mouth disease outbreak has the highest indirect costs and largest reduction in GHG emissions as it decreases the production of cattle as consumers switch to lower emitting meat commodities. Conversely, African swine fever has the smallest reduction in GHG emissions, reflecting the more industrialised nature of pig farming. Our modelling approach opens a provocative debate around how compensation to producers supports restocking and how this relates to commitments to net zero farming. Overall, an exotic disease outbreak may trigger an opportunity to switch to lower emitting breeds or species if a more holistic, joined up approach were taken by Government.

## 1. Introduction

Ambitious targets have been set for reducing greenhouse gas (GHG) emissions globally. More than 120 countries have pledged a commitment to reaching net zero by 2050–2070 at the most recent UNFCCC 26th Conference in Glasgow [1,2]. Livestock, whilst a fundamental part of most agricultural economies, has been under particular scrutiny due to the high methane emissions from enteric fermentation and management of manure [3–8]. Animal disease outbreaks have been found to worsen the impact of GHG emissions through the loss of efficiency and outputs, as well as the increased use of inputs needed to recover from disease [9–11]. However, to date, the relationship between the dynamics of market change from these disease outbreaks and the subsequent impact on emissions have been ignored.

A disease outbreak will directly reduce livestock numbers and increase production costs but will also have indirect impacts [12]. Direct costs, such as livestock mortality and treatment costs, are incurred during an outbreak, from first notification to eradication on the farm. These are likely to increase GHG emissions of livestock farming through reduced biological efficiency and increased waste [13]. Indirect costs are incurred in affected commodity markets and in other sectors after disease freedom is declared [14]. These include impacts on substitute or complementing agricultural markets [15]. Ignoring indirect cost may significantly underestimate the economic impact of an outbreak as these tend to be larger than direct costs [16]. However, a further hidden cost of the indirect effects is the impact of switching consumption between commodities on carbon emissions. Livestock commodities have highly varied carbon footprints with beef production consistently being identified as the highest emissions of the livestock sector. Conversely, a number of commentators have supported a switch to poultry meat as the main source of animal protein due to its industrialised nature and lower emissions intensity [17].

A small number of studies have included indirect economic impacts to disease outbreaks. [12,18] used partial equilibrium models to estimate the market level impacts of foot and mouth disease in the UK, finding indirect costs to be a substantial portion of overall costs. [19] conducted an ex-ante assessment of the direct and indirect costs of bluetongue on Scottish producers and commodity markets. Several studies have also estimated wider economy impacts of animal disease outbreak. [20,21] have used general equilibrium modelling and both found a wider economy impact of foot and mouth disease in Zimbabwe and Ireland, respectively. To date no studies have been conducted which link indirect economic costs with carbon impacts. The few studies which have looked at the climate consequences of animal disease outbreaks have only focused on the direct effect of deteriorated animal health on GHG emissions [13,22,23]. This is a significant oversight given the pressure on food systems to reduce carbon emissions and government commitments to meet Net Zero carbon target.

The purpose of this paper is to fill this gap and we contribute to the literature in a number of ways. Firstly, we employ a methodology to assess the impacts of four economically important exotic diseases. Very rarely do studies extend beyond a single disease and we provide a comparative assessment of impacts across diseases and sectors using a consistent framework. Secondly, we couple these indirect costs with GHG impacts from the estimated changes in consumption expected from these outbreaks. Finally, we estimated the economic value of carbon impacts. This allows us to examine any potential trade-offs between the indirect costs and carbon impacts of animal disease outbreaks using similar metrics. This offers a novel extension to the current literature and widens discussions on the GHG burden of livestock disease [24,25]. Our approach aims to support policy trajectories that promote net zero, especially in livestock dominated agricultural economies where animal health has been identified as a major intervention point for reducing emissions [26].

The four animal diseases considered in our analysis are: African swine fever, sheep pox, bluetongue, and foot and mouth disease. These diseases are classified as high-risk diseases by the World Organisation for Animal Health (WOAH) [27]. African swine fever and foot and mouth disease are highly contagious viral diseases that can led to high mortality rate. The latter affects a range of livestock (e.g. cattle, sheep, pigs, goats) while the former is found in domestic and wild pigs [28]. Bluetongue is a vector-borne viral disease that infect a range of ruminants and leads to mortality. Sheep pox is a viral disease characterized by widespread skin eruptions which also lead to animal mortality. Example of outbreaks of these diseases have already occurred in high income countries such as the UK, or in one or more of the neighbouring countries, and therefore there is a high probability that future incursions could occur [29–31]. Italy and Germany have recently experienced recent outbreaks of African swine fever while sheep pox has been reported in Cyprus and Spain in 2022–2023 [32]. In the UK, there was previous outbreaks of foot and mouth disease and bluetongue that occurred in 2001 and 2008 [33,34].

We apply our analysis to Scotland, as an example of a developed country with a heavy reliance on livestock activity as part of its agricultural economy. The ruminant sector (i.e., beef and lamb) has a high export value and the majority of agricultural land in Scotland is designated as grazing land [35,36]. The livestock sector is one of the main emitters of GHG in the Scottish economy generating 4.7 million tonnes of CO_2_e per year [37]. More pertinently Scotland has one of the most ambitious climate targets in the World. The Scottish Climate Change Act committed Scotland to meet net zero by 2045, ahead of other UK regions and other countries globally [38]. This is in parallel with an aim to significantly increase the value of its output by 2030 to meet the target set by Scotland’s food and drink partnership [39]. Increasing output value and reducing GHG emissions simultaneously therefore poses a significant challenge to the livestock sector.

## 2. Methods

### 2.1 Time series analysis

An exotic disease outbreak is expected to disrupt livestock markets by decreasing the domestic supply of livestock products, whilst increasing the supply of substitute products. Consequently, prices also change to achieve market equilibrium between supply and demand of affected products. A time series model fitted to historical data predicts the magnitude of change in market prices and quantities; and based on these predictions changes in market revenues are then estimated. We define these effects as “indirect economic costs” [12].

#### Data collection and transformation

Historic monthly time step price data were collected from various public and private sources 35, 36]. This is shown in Table 1 and includes 84 observations representing monthly producer price and quantity data of five Scottish livestock and feed markets (i.e. Cattle/beef, sheep/lamb, pigs/pork, poultry/chicken, and wheat feed) and available between January 2012 –December 2018. We adjusted for inflation and seasonality using the appropriate monthly producer price index. The base year was chosen as 2016 which is the year assumed for the hypothetical outbreak to eventuate. To remove seasonality from our data set, we first determined the pattern of the seasonality in our data by decomposing it into seasonal, trend, and residual components. The seasonal part was then removed additively and subsequently the data was log transformed to have a consistent scale across all data set variables.

**Table 1:** Summary statistics of input data used in the time series model.

Variable	Min	Median	Mean	Max	Standard Deviation
***Prices–output markets***					
Producer price of beef (£ per ton)	3,024	3,329	3,344	3,627	144
Producer price of pork (£ per ton)	1,096	1,418	1,396	1,582	121
Producer price of lamb (£ per ton)	3,137	3,897	3,943	5,199	434
Producer price of chicken (£ per ton)	127	142	143	187	9
***Quantity–output markets*** ^1^					
Quantity of cattle slaughtered (ton)	12,033	14,187	14,764	17,468	1,622
Quantity of pig slaughtered (ton)	611	2,349	2,614	6,069	985
Quantity of sheep slaughtered (ton)	2,073	5,009	4,800	7,106	1,122
Quantity of chicken slaughtered (ton)	1,841	6,111	6,542	12,254	2,536
***Price & output–input market***					
Price of feed wheat (£ per ton)	97	134	136	204	27
Quantity of feed wheat (ton)	11,656	15,279	15,470	19,832	1,976

^1^ Carcass weight; quantity of cattle slaughtered includes finished and culled cattle; quantity of pigs slaughtered includes sows and boars; and quantity of sheep slaughtered includes lambs and ewes. Foetuses lost as a result of abortions are not included in the reported output quantities.

#### Model specification

We initially applied the Augmented-Dickey-Fuller (ADF) and Phillips-Perron (PP) tests to inspect stationarity and the order of integration at which the time series data become constant. We then applied the Elliott–Rothenberg–Stock (ERS), Zivot-Andrews (ZA) and Lee-Strazizich LM tests to address any shortfalls in the results of the ADF and PP tests.

Cointegration represents long-term relationships between data series, which is identified when a stationary linear relationship between those variables could be established. Several unit residual-based root tests are available to examine cointegration in time series data [40]. We used Johansen trace Test to examine the presence of cointegration in our data set as it can detect multiple cointegrating vectors. Moreover, employing a time series model with minimum prediction inaccuracy is crucial for estimating the magnitude of the impacts incurred by an animal disease outbreak. The accuracy of model predictions was measured using the Mean Absolute Percentage Error (MAPE) and Theil’s inequality coefficient U [41].

#### Indirect economic impact assessment

The vector error correction model (VECM) was identified as the most suitable specification after conducting the above tests. VECM is a modified version of the Vector Autoregressive Model (VAR) which can be applied to integrated multivariate time series data [12,42,43]. The VECM produces an impulse response function (IRF), which is comparable to elasticities in standard econometric models. IRF estimates the degree of deviation between the original path and the new path generated from a shock (i.e. an animal disease outbreak); and the duration until the system stabilises at equilibrium (which could be its original path or a new level). In particular, the IRF, in logged form, quantifies the percentage change in one variable as a result of one percent change in another variable, e.g. on production quantities. VECM estimates the cumulative impact, as the sum of all the intermediate changes across the period of animal disease outbreak. Using VECM we therefore could calculate the total cumulative impact (expressed in percentage) that result from a one percent reduction in meat supply, due to culled or lost animals from disease outbreak. In addition, using VECM we were able to estimate the duration that the impact of this outbreak will take to dissipate. Three hypothetical outbreak scenarios were incorporated in our analysis, which assumes that 5, 20, and 35 percent of the total herd is lost or culled. These three scenarios represent small, medium, and large ranges which help in providing the likely range of impacts from a potential outbreak.

### 2.2 Accounting for GHG emissions

Changes in GHG emissions due to a disease outbreak were quantified by estimating the changes in the supply of commodities and then multiplied by emissions factors, where these represent the amount of GHG emitted per kg of meat [13; Table 2). Furthermore, to value the emissions from changes in market supply, we multiply the estimated changes in GHG by a carbon price. The UK Emissions Trading System (ETS) is a cap-and-trade system that was established in 2005 [44]. The ETS determines the maximum amount of GHG that could be emitted from the participating sectors. Implicitly the ETS price reflects the marginal abatement cost of meeting carbon targets from the sectors included in the ETS. As agriculture is not yet included in the ETS, we used the non-traded carbon price in our analysis. In 2022, the UK ETS price for non-traded sectors was estimated at £66 per tCO_2_e [44,45].

**Table 2:** Emission intensity factors used to quantify GHG emissions from changes in livestock supply due to disease outbreak.

Market	Emissions factors (kg CO_2_e/kg meat)
Beef^1^	26.0
Pork^2^	6.5
Lamb^3^	23.6
Chicken^4^	3.6
Feed wheat^5^	1.1

^1^ [46].

^2^ [47].

^3^ [48].

^4^ [49].

^5^ [50].

## 3. Results

### 3.1 Model testing

The presence of stationarity and the order of integration in the data series were initially examined by the ADF and PP tests. The results of these tests are outlined in Table 3. While the stationarity requirement for VECM is to have its variables integrated in order of one [I(1)], it is generally acceptable that time series with three or more variables only needs two of its variables to be in the order of one [51]. The ADF and PP tests showed that all variables are integrated in order of one except the “producer price of pork” variable which is shown to be integrated in order of two [I(2)]. To further examine this variable, we applied the ERS, ZA and Lee-Strazizich LM tests. As the ADF tend to have low power properties, ERS can improve the power properties of the ADF by using generalized least squares (GLS) [52,53]. In contrast to the ADF, the ERS showed that the “producer price of pork” variable is integrated in order of one. Failing of the ADF and PP tests to identify the “producer price of pork” variable as stationary could be due to the presence of outliers or structural breaks in the time series. Because of that we applied ZA and Lee-Strazizich LM tests which allow for one and two breaks, respectively. While ZA test result did not reject non-stationarity, Lee-Strazizich LM test rejected it, indicating that the “producer price of pork” variable is stationary and integrated in order of one when accounting for two breaks.

**Table 3:** Results of the Augmented-Dickey-Fuller (ADF) and the Phillips-Perron (PP) tests to determine the presence of stationarity and the order of integration at which the time series data become constant^1^.

Variable	ADF I(2)	ADF I(1)	ADF I(0)	PP I(2)	PP I(1)	PP I(0)
Producer price of beef	0.01	0.01	0.26	0.01	0.01	0.16
Producer price of pork	0.01	0.38	0.41	0.01	0.08	0.84
Producer price of lamb	0.01	0.01	0.07	0.01	0.01	0.01
Producer price of chicken	0.01	0.01	0.30	0.01	0.01	0.01
Price of feed wheat	0.01	0.02	0.85	0.01	0.01	0.86
Quantity of cattle slaughtered	0.01	0.01	0.07	0.01	0.01	0.01
Quantity of pig slaughtered	0.01	0.01	0.28	0.01	0.01	0.02
Quantity of sheep slaughtered	0.01	0.01	0.36	0.01	0.01	0.01
Quantity of chicken slaughtered	0.01	0.01	0.50	0.01	0.01	0.47
Quantity of feed wheat	0.01	0.01	0.85	0.01	0.01	0.19

^1^A p values < 0.05 means that the time series is stationary at the indicated order of integration.

Cointegration was also inspected using a Johansen Test (Table 4). For producer prices the test statistic exceeded the five percent critical value when testing for no cointegration (r = 0), one cointegration (r = 1), and two cointegrations (r = 2). This means that a linear combination of two time series was needed to achieve stationarity. Based on these findings, we therefore included the identified cointegration rank for producer prices in our VECM.

**Table 4:** Results of the Johansen Test that was used to examine cointegration in the time series data^1^.

Null hypothesis	Alternative hypothesis	Trace statistic	Critical value		
		Producer	10%	5%	1%
r ≤ 4	r ≥ 5	69.67	97.18	102.14	111.01
r ≤ 3	r ≥ 4	106.76	126.58	131.70	143.09
r ≤ 2	r ≥ 3	153.89	159.48	165.58	177.20
r ≤ 1	r ≥ 2	205.83	196.37	202.92	215.74
r = 0	r ≥ 1	273.62	236.54	244.15	257.68

^1^If trace statistic > critical value, then the null hypothesis is rejected.

To select the best specification for our data series, we compared the forecasting accuracy of standard VECM, VECM with outlier dummies, and VECM with structural breaks using MAPE and Theil’s tests. MAPE test results showed that VECM with outlier dummies has a superior outcome (MAPE prediction inaccuracy estimated at 1.03%) compared to standard VECM (inaccuracy of 1.09%) and VECM with structural breaks (inaccuracy of 1.96%). Theil’s test also showed similar results where VECM with outlier dummies has better prediction accuracy than standard VECM and VECM with structural breaks. We therefore estimate VECM with outlier dummies as our preferred specification.

### 3.2. Indirect economic costs

Across the different outbreak scenarios, the indirect costs were estimated to incur between £1 and £53 million (Table 5). Foot and mouth disease led to the largest adverse impacts among all the diseases considered in our analysis, which was estimated to range between £4 and £53 million, depending on the size of the outbreak scenario. These agree in magnitude with a similar study by [12], albeit with our updated values. This was followed by bluetongue (£1.6 – £11.3 million), sheep pox (£1.5 – £10.3 million), and African swine fever (£1 – £6.9 million). The magnitude of the costs of these diseases were highly dependent on the number of hosts that the disease could infect as well as the value and the size of their markets.

**Table 5:** Indirect costs of animal disease outbreak assuming a small, medium and large sizes of an outbreak (£ million).

Disease	Small (5%)	Medium (20%)	Large (35%)
African swine fever	-0.99	-3.95	-6.90
Sheep pox	-1.55	-6.03	-10.26
Bluetongue	-1.67	-6.58	-11.36
Foot & mouth disease	-4.17	-23.52	-53.14

The estimated costs reported above represent the net impact on the four livestock markets. For all animal diseases, losses in the main host market were offset by gains in other markets. This is a result of consumers switching between livestock commodities due to changes in supply and consequent price shifts. The degree of offset is however different from one disease to another. Table 6 shows the impacts of a medium size outbreak in the main host livestock market only and in all commodity markets altogether (the main host markets, alternative livestock markets, and feed market) for each animal disease. For instance, the impact of a medium size outbreak of sheep pox on the lamb market is estimated to be approximately £13 million. This impact was reduced to approximately £6 million if we consider the benefits to other livestock markets from consumer switching, i.e. gains in other markets such as beef and chicken markets partially offset losses incurred in the lamb market (~ £7 million). Similarly, half of the losses of bluetongue and foot and mouth disease have also been offset by gains in other markets. Conversely, only a small portion of losses (~£300 thousand) caused by African swine fever in the pig/pork market has been offset by minor gains in other markets, particularly the cattle market. This is due to beef and lamb not being a perfect substitute for pig meat as their prices are much higher.

**Table 6:** Indirect costs, in £ million, of a medium size outbreak in the main host livestock market only and all livestock commodity markets altogether (the main host markets, substitute/complement livestock markets, and feed market) for each animal disease.

Disease	Host species	Cost in all markets (£ million)	Cost in main host market (£ million)
African Swine fever	Pigs	-3.97	-4.31
Sheep pox	Sheep	-6.06	-13.68
Bluetongue	Sheep		-13.68
Bluetongue	Cattle		-3.28
Bluetongue	Total	-6.61	
Foot & mouth disease	Cattle		-44.25
Foot & mouth disease	Pigs		-4.31
Foot & mouth disease	Sheep		-13.68
Foot & mouth disease	Total	-23.52	

**Fig 1:**
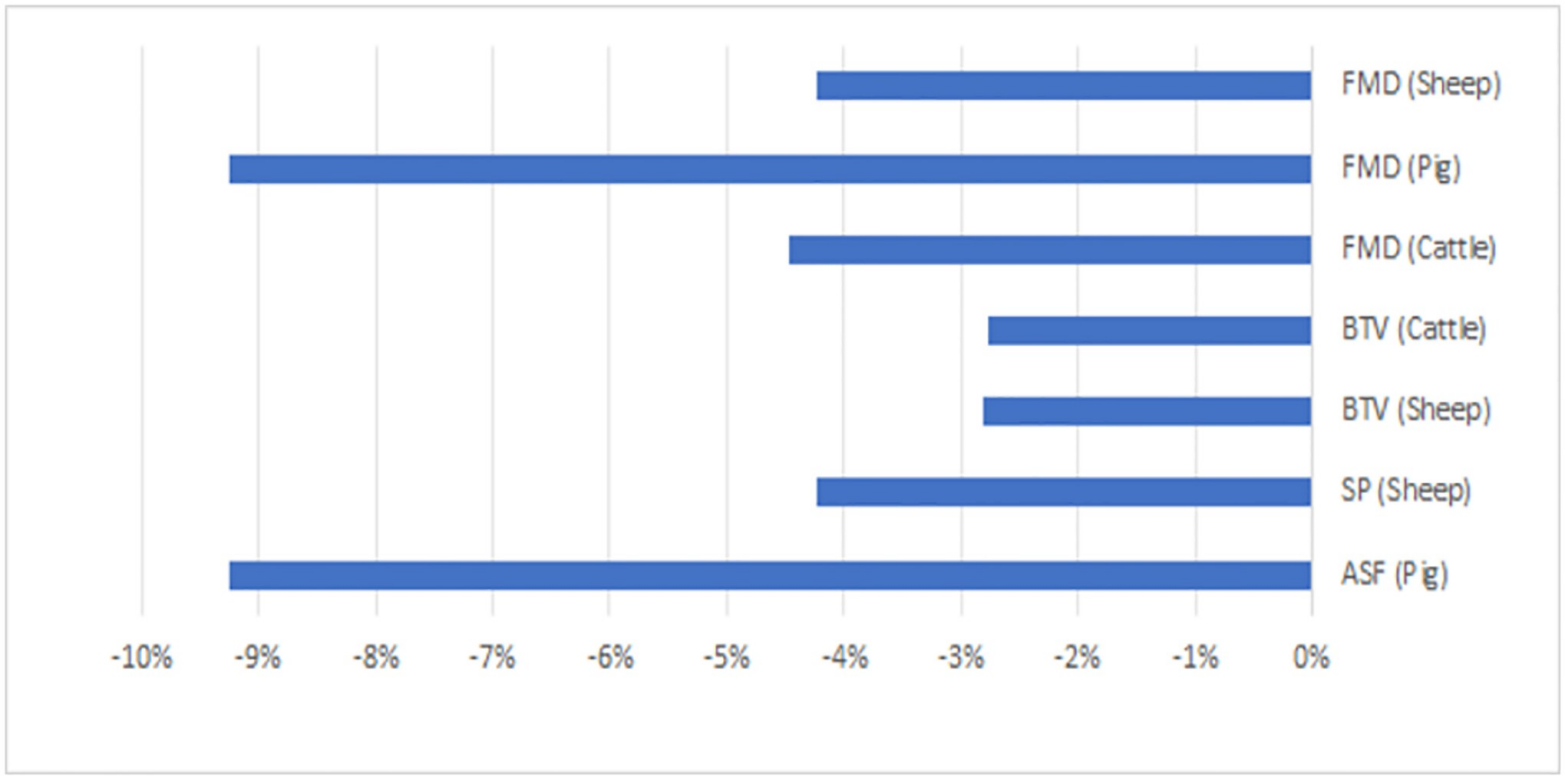
The value of the estimated impulse response function (IRF) in the main affected livestock market (percentage of reduced supply due to 1% of culled or lost animals).

The Impulse response function (IRF), which shows the relative response (in percentage terms) to a one percent supply shock, on the main host and closely related markets, is shown in Fig 1. Our results show that pig producers react less favourably to culling of pigs in the market compared to sheep, chicken, and cattle producers’ (Fig 1). For instance, the IRF for pig producers [-9.24] is higher than the IRF estimated for sheep [-4.23] and cattle [-4.47] producers. This means that for every one percent of lost or culled pigs due to a disease outbreak, the pig producers will reduce their market supply by ~9%, while for every one percent of lost or culled sheep or cattle, these producers will reduce their market supply by ~4%. In general, reduction in market supply was higher than the direct impact of lost or culled animals because some producers, for instance, might decide to switch to an alternative livestock market which is not threatened by an animal disease outbreak.

**Fig 2:**
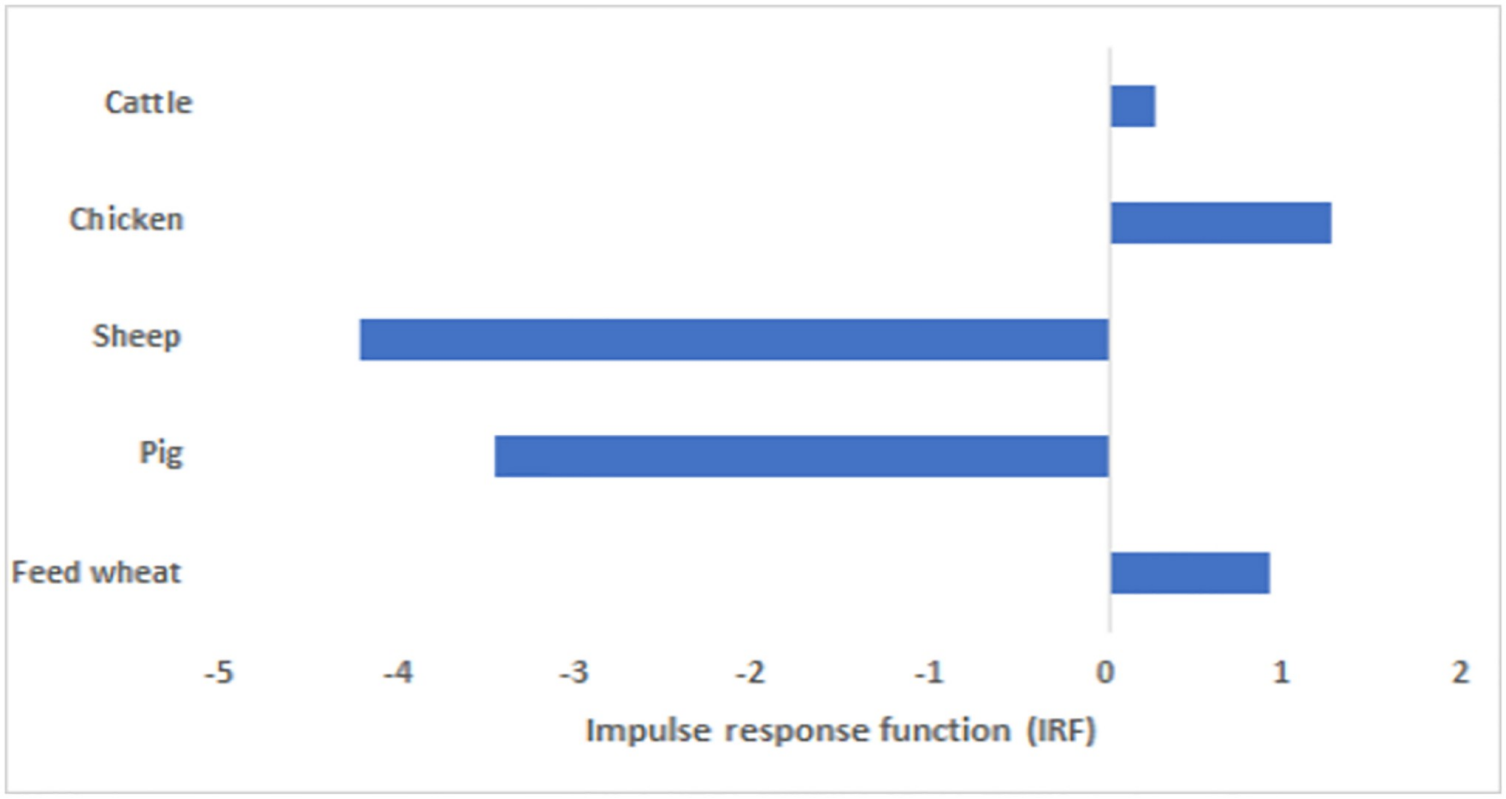
The Impulse response function (IRF) for a sheep pox outbreak, showing the cumulative percentage change in livestock and feed supply associated with 1% increase in sheep culled.

There is also an impact on the supply and prices of the substitute and complementary markets, the magnitude of which is captured by the IRF. For instance, a sheep pox outbreak will reduce the supply of lamb (4.2%) and pork (3.4%) but will increase the supply of beef (0.3%) and chicken (1.2%) as producers respond to more favourable prices within their sector. Producer prices have generally increased either due to reduced supply or increased demand (Fig 2).

### 3.3. Indirect GHG emissions

Depending on the disease and size of the outbreak, all modelled diseases lead to a net reduction in GHG emissions ranging from 5 and 668 thousand tonnes CO_2_e (Table 7). An outbreak of foot and mouth disease leads to the largest reduction in GHG emissions, as this decreases the production of cattle, pig, and sheep and only increases the production of chickens. Conversely, African swine fever led to the smallest reduction in GHG emissions as there were only minor shifts to other markets.

**Table 7:** Indirect climatic effects (changes in GHG emissions) due to animal disease outbreak (1000 tCO_2_e).

Disease	GHG (1,000 tCO_2_e)		
	Small (5%)	Medium (20%)	Large (35%)
African swine fever	-5	-22	-38
Sheep pox	-16	-64	-112
Bluetongue virus	-48	-191	-335
Foot & mouth disease	-95	-382	-668

Using the ETS non-traded carbon values, the UK GHG reductions, dependant on the severity of outbreak and commodities, were valued between £0.4–£44 million (Table 8). In the medium outbreak scenario, the value of reduced emissions due to a foot and mouth disease outbreak, for instance, was estimated at £25 million, while the value of reduced emissions due to African swine fever was £1.4 million.

**Table 8:** Valuation of the changes in GHG emissions from animal disease outbreak using ETS carbon price and social cost of carbon (£ million).

Disease^1^	ETS^2^		
	Small (5%)	Medium (20%)	Large (35%)
ASF	0.4	1.4	2.5
SP	1.1	4.2	7.4
BTV	3.2	12.6	22.1
FMD	6.3	25.2	44.1

^**1**^ ASF: African swine fever; SP: Sheep pox; BTV: Bluetongue; FMD: Foot & mouth disease.

^**2**^ Carbon price for non-traded sectors in the UK emissions trading scheme (estimated at £66 per tCO_2_e).

## 4. Discussion

A growing body of literature is focusing on the global burden of livestock disease and its role in managing climate policy [10,54,55]. Ostensibly diseases will lower efficiency levels, incur higher or wasted input use and early mortalities which leads to calls for optimal livestock management [22,56]. Conversely, a growing body of opinion has been directed towards the role livestock has within agricultural economies to meet climate targets [56–58]. These debates tend to be more global and ignore the individual economic impacts of significant structural change [7]. Our study assesses the threat of a number of significant diseases at a detailed scale which will—if experienced in isolation—lead to economic losses for the agricultural economy but, perversely, support reductions in GHG emissions. As such it provides a localised empirical response to the observation of [55] that there will be an increase in the supply of a livestock sector affected by disease. [59] challenge the simplification of this assumption and our neo-classical economic framework provides evidence that other sectors benefit from animal disease outbreaks more than the sector affected.

### 4.1. Comparative analysis of indirect costs

Our study suggests that indirect costs differ based on the severity of outbreak, the sector which experiences that outbreak and the related sectors that offer substitutes for consumption. An animal disease outbreak will reduce the supply of the affected meat product leading to an increase in its price and consumption switching to other relatively cheaper meat alternatives. The four diseases analysed range in severity based on current structures and production systems of affected livestock commodities but could also be considered in the lower bound of estimates as we do not include direct costs, though the magnitude of cost shares will be similar based on previous outbreaks [60]. Moreover, this shows that not accounting for the indirect cost of a disease leads to significant underestimation of impact. For instance, an outbreak of foot and mouth disease was estimated to incur an indirect cost of approximately 4–10 percent of the beef sectors value to the Scottish economy [12]. Our results support the contention that these diseases pose a significant economic impact for the livestock sector and, if indirect costs are included within impact assessments, would offer more justification for increased investment in prevention measures such as surveillance, traceability systems and diagnostic capabilities for these and other high-risk diseases.

### 4.2. The indirect role of animal health in greenhouse gas emissions

Our analysis showed that animal disease outbreaks could indirectly lead to reduction of GHG emissions, depending on the disease type and the size of the outbreak, which was estimated to range between 0.05–0.67 million tonnes. This is a tangible and positive amount for GHG abatement. Relative to the two key mitigation options that support Scotland’s agriculture’s net zero target [61], afforestation and changes in farming practices, a severe foot and mouth disease outbreak is equivalent to approximately 11% of the afforestation target and 23% of the farming management options of 6 and 2.9 million tonnes [62,63]. The abatement in GHG emissions due to a disease outbreak could be valued between £0.3 – £44 million. This means that the indirect benefits gained from reduced emissions could match the indirect costs of an outbreak, depending on the size of the outbreak and the disease type.

The response of producers to a disease outbreak however is often to restock, and this is driven in part by compensation schemes for these diseases [64,65]. This places focus on compensation payments to support returning to business to a pre-disease state. The diseases we have outlined are potentially devastating for farming communities, but Government compensation is offered to encourage positive biosecurity behaviours [66]. Accordingly, our analysis should mediate discussions around the underlying rationale for disease compensation payments when a net zero route is prioritised. The desire to restock may therefore need to be attached to differing terms for compensation or lower or no compensation [64,65]. In the least it argues for a more holistic approach between animal disease compensation payments and incentives for reducing GHGs. Moreover, switching consumption will also influence the diet agenda in terms of nutrients gained from access to specific protein sources, or induced health damages, e.g. red meat consumption, from switching consumption to maintain protein levels [17]. Arguably, then, it is not worth discounting that a disease shock offers a desirable, if potentially inequitable, outcome for societal goals.

### 4.3. Limitations

Our findings provide better understanding of the trade-offs between multiple indirect effects of animal disease outbreak which could be used to inform the public and private sector on animal health and climate change policies. However, whilst we focus our analysis at the Scottish level, we have discounted trade between countries predominantly due to the lack of equivalent time series of export and import data. Currently Scotland is mostly self-sufficient in pork, beef, and lamb; but it is not inconceivable that the gap left from loss in home supply is delivered through imported meat. This potentially has larger implications for off-setting emissions lost through replacement meat produced to lower environmental standards. Accordingly, a further area for research would be dynamic trade modelling, which may come at a cost for resolution of understanding micro-level impacts discussed here. Nevertheless, while most of the Scottish exports are directed to the rest of the UK, significant amounts are also exported to the rest of the world. For instance, Scottish beef and lamb annual exports were valued at £39 and £34 million, representing 6 and 26 percent of total Scottish production, respectively [67]. This consequently indicates that the quantified benefits to Scottish producers who offer substitutes to the affected products may be overestimated and the quantified indirect costs in our analysis underestimated.

A further route to investigation would be the economy-wide effects of a radical change in compensation policy. As there are multipliers between agriculture and rural industries, any change in production may influence revenues of related industries, employment and livelihood consequences. The livestock sector is a key contributor to the Scottish economy, representing approximately 40 percent of total agricultural output with an estimated value of £1.6 billion [35]. These means that although an outbreak might have benefits in indirectly reducing GHG emissions, it will also lead to significant indirect costs not only on the livestock sector but also on the whole economy. Accordingly, establishing a just transition for these communities is a principal aim of society [68] and our approach, though provocative, offers one route to exploring the consequences further.
